# Prevalence, indications, and outcomes of caesarean section deliveries in Ethiopia: a systematic review and meta-analysis

**DOI:** 10.1186/s13037-020-00236-8

**Published:** 2020-04-07

**Authors:** Getnet Gedefaw, Asmamaw Demis, Birhan Alemnew, Adam Wondmieneh, Addisu Getie, Fikadu Waltengus

**Affiliations:** 1 Department of Midwifery, College of Health Sciences, Woldia University, P.O.Box:400, Woldia, Ethiopia; 2 Department of Nursing, College of Health Sciences, Woldia University, P.O.Box:400, Woldia, Ethiopia; 3 Department of Medical Laboratory Sciences, College of Health Sciences, Woldia University, P.O.Box:400, Woldia, Ethiopia; 4 Department of Midwifery, College of Medicine and Health Sciences, Bahir Dar University, Bahir Dar, Ethiopia

**Keywords:** Caesarean section, Maternal complications, Meta-analysis, Neonatal complications

## Abstract

**Background:**

Caesarean section rates have increased worldwide in recent decades. Caesarean section is an essential maternal healthcare service. However, it has both maternal and neonatal adverse outcomes. Therefore this systematic review and meta-analysis aimed to estimate the prevalence, indication, and outcomes of caesarean section in Ethiopia.

**Methods:**

Twenty three cross-sectional studies with a total population of 36,705 were included. Online databases (PubMed/Medline, Hinari, Web of Science, and Google Scholar) and online university repository was used. All the included papers were extracted and appraised using the standard extraction sheet format and Joanna Briggs Institute respectively. The pooled prevalence of the caesarean section, indications, and outcomes was calculated using the random-effect model.

**Result:**

The overall pooled prevalence of Caesarean section was 29.55% (95% CI: 25.46–33.65). Caesarean section is associated with both maternal and neonatal complications. Cephalopelvic disproportion [18.13%(95%CI: 12.72–23.53] was the most common indication of Caesarean section followed by non-reassuring fetal heart rate pattern [19.57% (95%CI: 16.06–23.08]. The common neonatal complications following Caesarean section included low APGAR score, perinatal asphyxia, neonatal sepsis, meconium aspiration syndrome, early neonatal death, stillbirth, and prematurity whereas febrile morbidity, surgical site infection, maternal mortality, severe anemia, and postpartum hemorrhage were the most common maternal complications following Caesarean section.

**Conclusion:**

In this systematic review and meta-analysis, the rate of Cesarean section was high. Cephalopelvic disproportion, low Apgar score, and febrile morbidity were the most common indication of Caesarean section, neonatal outcome and maternal morbidity following Caesarean section respectively. Increasing unjustified Caesarean section deliveries as a way to increase different neonatal and maternal complications, then several interventions needed to target both the education of professionals and the public.

## Background

Caesarean section is the commonest operative delivery technique in the world. Caesarean section is the delivery of the fetus, membrane, and placenta through abdominal and uterine incision after fetal viability [1].

The rate of Caesarean section is different across countries even between urban and rural areas, due to different socio-economic statuses, and opportunities to access public and private health care services [2].

According to American College of Obstetricians and Gynecologist (ACOG) report, Caesarean delivery significantly increased woman’s risk vulnerability of pregnancy-related morbidity and mortality which accounts (35.9 deaths per 100,000 live deliveries) as compared to a women posses vaginal delivery (9.2 deaths per 100,000 live births) [3].

Despite Caesarean section a life saving medical intervention and procedures to the decrease adverse birth outcome, controlling different postoperative neonatal and maternal complications are challenging in terms of patient safety, long duration of hospital stay, cost and psychological trauma. Maternal outcomes of Caesarean section included: postpartum fever, surgical site infection, puerperal sepsis, maternal mortality whereas neonatal sepsis, early neonatal death, stillbirth, perinatal asphyxia, low Apgar score, and prematurity were the most common complication of the newborn [4–6].

Despite World Health Organization (WHO) recommended the optimal rate of Caesarean section should be lie between 5 and 15%, it is significantly increasing even if the reasons for the continued increase in the Caesarean rates are not completely understood: women are having fewer children, maternal age is rising, use of electronic fetal monitoring is widespread, malpresentation especially breech presentation, frequency of forceps and vacuum delivery is decreased, rate of labor induction increases, obesity dramatically rises and Vaginal birth after Caesarean decreased are some of the possible explanations [7].

Previous Caesarean scar, malpresentation and malposition, antepartum hemorrhage, obstructed labor, cephalopelvic disproportion, non-reassuring fetal heart rate pattern, and multiple pregnancies are the most common indications of Caesarean section [4–6, 8].

According to the 2016 Ethiopia Demographic and Health Survey, the rate of C-section (21.4%) in Addis Ababa was far more than the 10–15% rate recommended by the world health organization. EDHS (2016) report showed there is an absolute difference rate of Caesarean section across different regions in Ethiopia which accounts Amhara (2.3%), Oromia (0.9%), SNNPR(1.9%), Afar (0.7%), Tigray (2%), Somali(0.4%), Benishangul Gumuz (1%) too far from the lowest 5% WHO recommendation of Caesarean section deliveries. This review helps to see C-section rates beyond 15% and below 5% is considered medically unjustified or unnecessary, with negligible benefits for most mothers, and yet costly and unequally distributed throughout the population [9, 10].

Ethiopia is a good case study to assess Caesarean prevalence, indications, and outcomes because, like other countries in sub-Saharan Africa, maternal mortality and neonatal mortality did not decline sufficiently to meet the Sustainable Development Goal for maternal health and child, and was estimated at 412 maternal deaths and 29 neonatal deaths per 100,000 live births in 2016 [9].

Despite a few single studies stated different maternal and fetal outcomes of Caesarean section, there is a lack of data to show the distribution and outcome of Caesarean section in different regions where they are provided.

This systematic review and meta-analysis aimed to estimate the pooled prevalence of Caesarean section deliveries and to determine the indications and outcomes of Caesarean section deliveries in Ethiopia.

## Methods

This systematic review and meta-analysis have been conducted to estimate the pooled prevalence of Caesarean section, indications, maternal and neonatal outcomes in Ethiopia via the standard PRISMA checklist guideline.

### Search strategy

International databases (PubMed, Google Scholar, Web of science and HINARI), different gray works of literature and articles in the university repository were included. The searching engine terms were used using PICO formulating questions. These are: “newborn”, “neonatal”, “birth outcome”, “stillbirth”, perinatal asphyxia””, “neonatal sepsis”, “prematurity”, “early neonatal death”, “low Apgar score”, “preterm”, “maternal mortality”, “wound infection”, “surgical site infection”, “febrile morbidity”,” puerperal sepsis”, “puerperal fever”, postpartum hemorrhage”, “blood loss”, “anemia”, “leading factors of Caesarean section”, “indications of Caesarean section”, “Ethiopia”. The following search engine terms were used: neonate OR newborn OR women OR infant OR fetal OR children AND “neonatal sepsis” OR “perinatal asphyxia” OR “low Apgar score” OR “stillbirth” OR “prematurity” OR “preterm birth” OR “early neonatal death” OR “perinatal” OR “neonatal death” OR “preterm “puerperal sepsis” OR “puerperal fever” OR “wound infection” OR “surgical site infection” OR “postpartum hemorrhage” OR “anemia” OR “maternal mortality” OR “maternal death” OR “blood loss” OR “indication of Caesarean section, ‘factors of Caesarean section”, “leading factors of Caesarean section”, “fetal indication of CS”, “Maternal indication of CS”AND Ethiopia and related terms.

### Inclusion and exclusion criteria

Twenty three (23) cross-sectional studies were included. Articles reported prevalence or/and an indication, or/and neonatal outcomes or/and maternal outcomes were incorporated. Only English language literature and research articles were included. Studies published till October 2019 were reviewed, screened and appraised for this study. Whereas, articles without full abstracts or texts and articles reported out of the scope of the outcome interest were excluded.

### Quality assessment

GG, AD & AW independently evaluated the quality of each study using the Joanna Briggs Institute (JBI) quality appraisal checklist [11]. Any disagreement was resolved by the hindrance of the third reviewer (FW, BA &AG). The following JBI items used to appraise cross-sectional studies were: [1] inclusion criteria, [2] description of study subject and setting, [3] valid and reliable measurement of exposure, [7] objective and standard criteria used, [9] identification of confounder, [10] strategies to handle confounder, [12] outcome measurement, and [13] appropriate statistical analysis. Hence, studies considered with the JBI checklist value of 50% and above of the quality assessment indicators as low risk and good to be included for the analysis.

### Data extraction

All the datasets are exported to Endnote version X8 software, and then we transferred to the Microsoft Excel spreadsheet to remove duplicate data in the review. Three authors (GG, AD, and AG) independently extracted all the important data using a standardized JBI data extraction format. Any disagreement between reviewers was resolved by the second team reviewers (FW, BA & AW). The consensus was declared through critical discussion and evaluation of the articles by the independent group reviewers. The name of the author, sample size, publication year, study area, response rate, region, study design, the prevalence of specific maternal outcomes, the prevalence of neonatal outcomes, indications of Caesarean section, and prevalence of Caesarean section with 95%CI were extracted.

### Outcome of measurements

#### Neonatal outcomes

Any neonatal outcomes reported following C-section (Stillbirth, prematurity, neonatal sepsis, perinatal asphyxia, low Apgar score, and early neonatal death) were included.

#### Maternal outcomes

Any maternal complications identified after C-section were included puerperal sepsis, wound infection (surgical site infection), febrile morbidity (puerperal fever), postpartum hemorrhage, severe anemia, and maternal mortality.

#### Indications of caesarean section

Both maternal and fetal indications (obstructed labor, cephalo pelvic disproportion, NRFHRP (Non-reassuring fetal heart rate pattern), multiple gestations, failed induction, malpresentation, malposition, and antepartum hemorrhage) were included.

### Data analysis

A Funnel plot and Eggers regression test was used to check publication bias [14]. Cochrane Q-test and I-squared statistics were computed to check the heterogeneity of studies [15, 16]. Pooled analysis was conducted using a weighted inverse variance random-effects model [17]. Subgroup analysis was done by study region (area), and sample size. STATA version 11 statistical software was used to compute the analysis. Forest plot format was used to present the pooled point prevalence, indications and outcomes of C-section with 95%Cl.

## Results

### Characteristics of the included studies

Four hundred twenty-three studies were retrieved at PubMed, Google Scholar, Science Direct, web of science, HINARI and other gray and online repository accessed articles regarding prevalence, indications, and the maternal and fetal outcome of Caesarean section in Ethiopia. After duplicates were expunged, 278 studies remained.

**Fig. 1:**
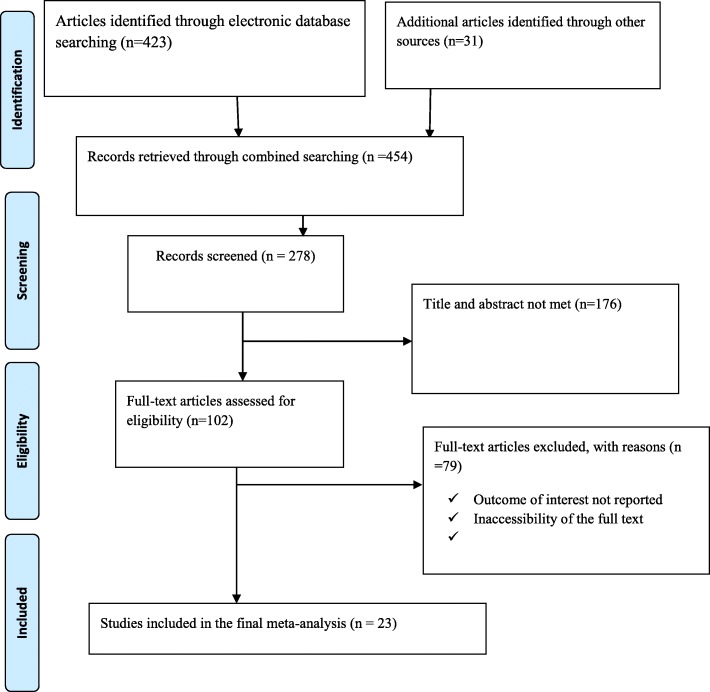
Flow chart of study selection for systematic review and meta-analysis of indications, maternal and fetal outcomes of cesarean section in Ethiopia

Out of 278 remained articles, 176 articles were excluded after review of their titles and abstracts. Therefore, 102 full-text articles were accessed and assessed for inclusion criteria, which resulted in the further exclusion of 79 articles primarily due to reason. As a result, 23 studies were met the inclusion criteria to undergo the final systematic review and meta-analysis (Fig. 1) (Table 1).

**Table 1:** Study characteristics included in the systematic review and meta-analysis

Authors	Region	Study area	Study design	Sample size	Prevalence	Response rate	Quality
Jebessa S et al. [12]	SNNPR	Attat	cross-sectional	3722	30.494	100%	Low risk
Bizuneh A, Ayana G [13]	SNNPR	Addis Ababa	cross-sectional	2345	24.819	100%	Low risk
Amanuel G et al. [18]	Tigray	Mekelle	cross-sectional	9348	31.14	100	Low risk
Tesfaye T et al. [19]	SNNPR	Sidama	cross-sectional	469	NA	100	Low risk
Tenaw Z, et al. [20]	SNNPR	Hawassa	cross-sectional	300	49.333	98.7	Low risk
Abayneh A [21]	Amhara	Gondar	cross-sectional	323	29.721	100	Low risk
Almaz H et al. [22]	SNNPR	Hawassa	cross-sectional	3195	17.089	100	Low risk
Ayodeji O et al. [23]	Addis Ababa	Addis Ababa	cross-sectional	411	63.747	100	Low risk
Hordofa G, Ashenafi S [24]	SNNPR	Mizan Aaman	cross-sectional	342	21.053	100	Low risk
Melaku K et al. [25]	Amhara	Finote selam	cross-sectional	2267	11.028	100	Low risk
Bago BJ et al. [26]	Hawassa	Hawassa	cross-sectional	422	35.071	98	Low risk
Abebe et al. [27]	Amhara	Bahirdar	cross-sectional	2967	24.368	100	Low risk
Hiwot et al. [28]	Addis Ababa	Addis Ababa	cross-sectional	298	38.255	100	Low risk
Alemayeu et al. [29]	Harar	Harar	cross-sectional	422	NA	100	Low risk
Bayou YT et al. [30]	Addis Ababa	Addis Ababa	cross-sectional	835	19.281	100	Low risk
Wondie et al. [31]	Amhara	Dessie	cross-sectional	512	47.656	98.4	Low risk
Tsega et al. [32]	Harar	Harar	cross-sectional	601	34.276	95.4	Low risk
Geremew et al. [33]	SNNPR	Attat	cross-sectional	5611	27.571	100	Low risk
Solomon et al. [34]	Oromia	Chiro	cross-sectional	407	18.182	100	Low risk
Taye and Yuya [35]	Oromia	Jimma	cross-sectional	388	28.351	100	Low risk
Mengesha et al [36]	Tigray	Mekelle	cross-sectional	338	NA	100	Low risk
Gebre S, et al. [37]	Tigray	Dansha	cross-sectional	749	13.218	100	Low risk
Melese et al. [38]	Amhara	Woldia	cross-sectional	433	30.947	100	Low risk

### Prevalence of caesarean section in Ethiopia

**Fig. 2:**
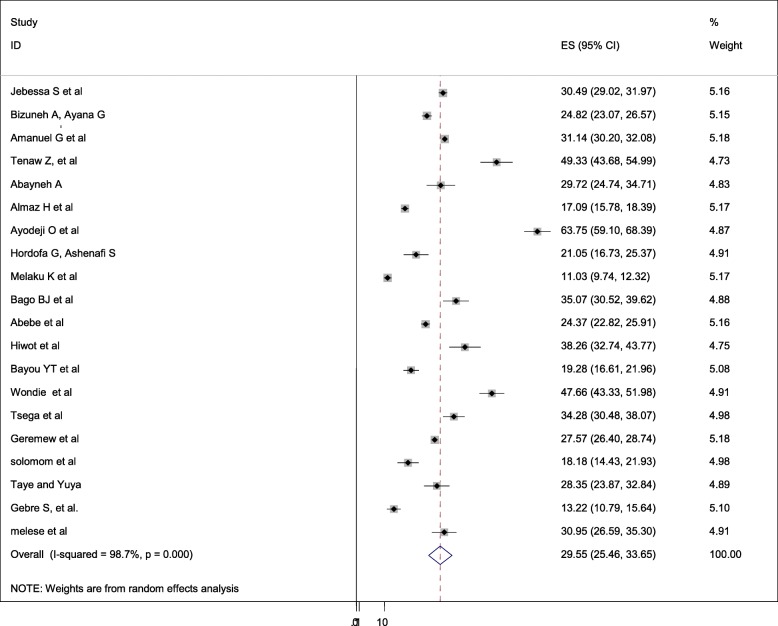
Forest plot of the pooled prevalence of cesarean section in Ethiopia

The overall pooled prevalence of Caesarean section is presented with a forest plot **(**Fig. 2**).** Therefore, the pooled estimated prevalence of Caesarean section in Ethiopia was 29.55% (95% CI; 25.46–33.65; I2 = 98.7%, *P* < 0.001).

### Publication bias

**Fig. 3:**
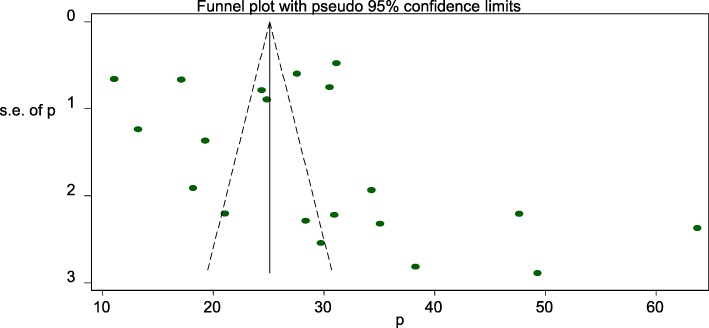
Funnel plot with 95% confidence limits of the pooled prevalence of cesarean section in Ethiopia

A funnel plot was assessed for the asymmetry distribution of the Caesarean section using visual inspection of the forest plot **(**Fig. 3**).** Egger’s regression test showed with a *p*-value of 0.251 indicated the absence of publication bias.

### Subgroup analysis

**Fig. 4:**
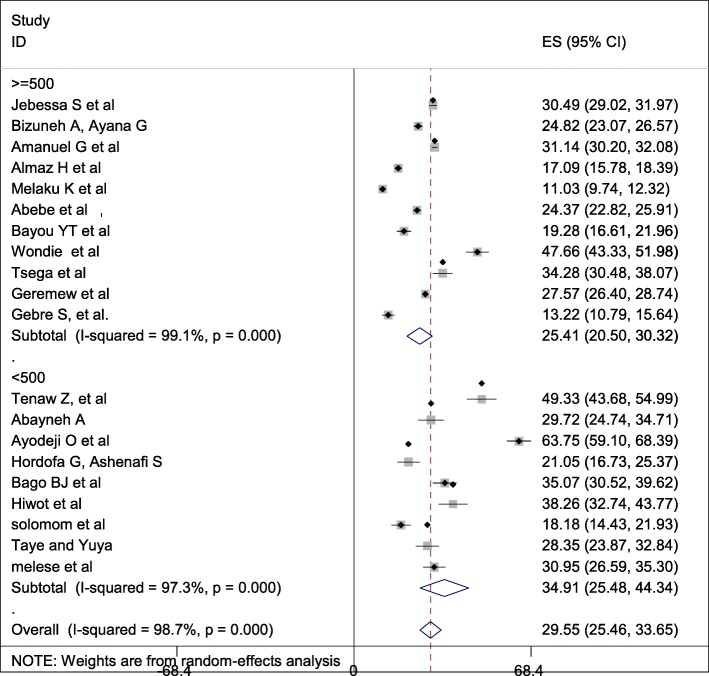
Forest plot of the subgroup analysis based on region (area) of the study

**Fig. 5:**
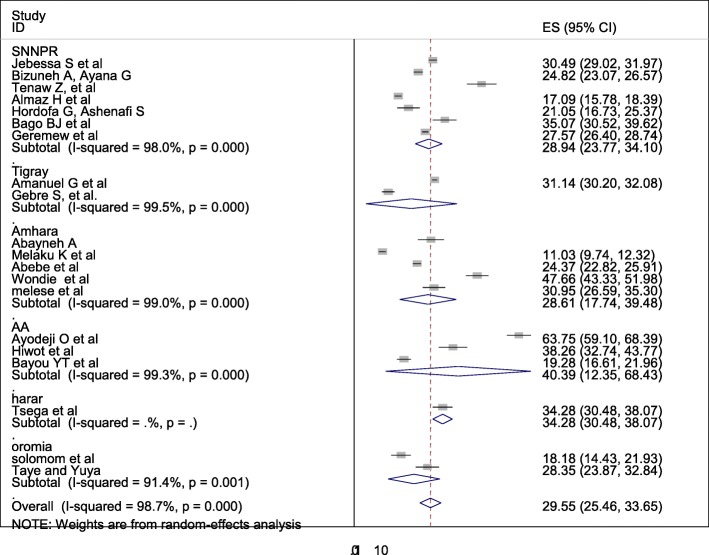
Forest plot of the subgroup analysis based on the sample size of the study

Subgroup analysis was computed with the evidence of heterogeneity. Hence the Cochrane I^2^ statistic =98.7%, *P* < 0.001) showed the presence of marked heterogeneity in this study. Therefore subgroup analysis was implemented using the study area (region) and sample size using random model effect analysis. Regarding the study area (region), the prevalence of Caesarean section was highest in Addis Ababa, accounted for 40.39% (95%CI: 12.35, 68.43) whereas the rate of Caesarean section was higher among studies of having sample size less than 500, accounted for 34.91% (95%CI: 25.48–44.34) **(**Fig. 4-5**).**

### Indication of caesarean section

In this systematic review and meta-analysis; obstructed labor, cephalopelvic disproportion, multiple pregnancies, non-reassuring fetal heart rate pattern (NRFHRP), failed induction and augmentation, malpresentation and malposition, and antepartum hemorrhage are the most common indications of Caesarean section. In this systematic review and meta-analysis, cephalopelvic disproportion (CPD) is the most common indication of Caesarean section followed by non-reassuring fetal heart rate pattern (NRFHRP), and obstructed labor in Ethiopia **(**Table 2**).**

**Table 2:** Indications of Caesarean section in Ethiopia

Indications of Caesarean section	Model	Status of heterogeneity	Prevalence (95%CI)	I^2^ (%)	*P*-value
Cephalopelvic disproportion	Random	Marked heterogeneity	18.13(12.72–23.53)	99.1	≤0.001
Obstructed labor	Random	Marked heterogeneity	15.25(5.21–25.3)	99.8	≤0.001
Failed induction/Augmentation	Random	Marked heterogeneity	6.38(4.53–8.23.3)	98.1	≤0.001
Non-reassuring fetal heart rate pattern (NRFHRP)	Random	Marked heterogeneity	19.57(16.06–23.08)	90.4	≤0.001
Antepartum hemorrhage	Random	Marked heterogeneity	7.59(6.1–9.08)	95.7	≤0.001
Malpresentation and malposition	Random	Marked heterogeneity	9.74(7.08–12.41)	98.6	≤0.001
Having more than one pregnancy (multiple gestations)	Random	Marked heterogeneity	5.17(4.08–6.25)	91.7	≤0.001

### Neonatal complication following caesarean section in Ethiopia

Among women who underwent Caesarean section; neonatal sepsis, early neonatal death, stillbirth, low Apgar score, perinatal asphyxia (PNA), meconium aspiration syndrome, and prematurity were the reported neonatal complications in this study. Among neonatal complications, low Apgar score was the most common adverse complication of the newborn followed by perinatal asphyxia and neonatal sepsis respectively in Ethiopia (Table 3).

**Table 3:** Neonatal complications following Caesarean section in Ethiopia

Neonatal complications	Model	Status of heterogeneity	Prevalence (95%CI)	I^2^ (%)	*P*-value
Prematurity	Random	Marked heterogeneity	8.26(2.81–13.7)	99.8	≤0.001
Low APGAR score	Random	Marked heterogeneity	22.21 (13.57–30.85)	98.6	≤0.001
Meconium aspiration syndrome (MAS)	Random	Marked heterogeneity	10.47(3.61–17.33)	98.8	≤0.001
Perinatal asphyxia (PNA)	Random	Marked heterogeneity	19.91(7.52–32.2)	99.9	≤0.001
Neonatal sepsis	Random	Marked heterogeneity	19.15(1.78–36.51)	99.9	≤0.001
Early neonatal death (END)	Random	Marked heterogeneity	2.19(0.98–3.37)	97	≤0.001
Stillbirth	Random	Marked heterogeneity	5(3.11–6.89)	97	≤0.001

### Maternal complications following caesarean section in Ethiopia

Following Caesarean section different adverse maternal complications were reported. Febrile morbidity, puerperal sepsis, postpartum hemorrhage, surgical site infection, maternal mortality, and severe anemia were the most common adverse maternal complications following Caesarean section. Puerperal fever or febrile morbidity was the leading cause of maternal morbidity following Caesarean section followed by postpartum hemorrhage in Ethiopia (Table 4**).**

**Table 4:** Maternal complications following Caesarean section in Ethiopia

Maternal complications	Model	Status of heterogeneity	Prevalence (95%CI)	I^2^ (%)	*P*-value
Maternal mortality	Random	Marked heterogeneity	0.66(0.14–1.17)	81.4	≤0.001
Severe anemia	Random	Marked heterogeneity	2.06 (0.04–4.09)	80.6	≤0.001
Puerperal fever or febrile morbidity	Random	Marked heterogeneity	16.44 (10–22.87)	99.9	≤0.001
Surgical site infection	Random	Marked heterogeneity	10.81 (5.74–15.88)	98.9	≤0.001
Postpartum hemorrhage	Random	Marked heterogeneity	13.25 (8.34–18.15)	99.3	≤0.001

## Discussion

Despite Caesarean section is an essential component of comprehensive obstetric and newborn care for reducing maternal and neonatal mortality, there is a lack of data regarding Caesarean section rates, its indications and outcomes in Ethiopia. Studies showed negative or no complications of Caesarean on neonatal mortality in low and middle-income countries where the Caesarean rates are high. Cesarean section is very crucial in settings where the Caesarean rates are very low, due to the unavailability of Caesarean [39].

Caesarean sections can cause significant and sometimes permanent complications, disability or death particularly in settings that lack the facilities and/or capacity to properly conduct safe surgery and treat surgical complications [40].

Low- and middle-income countries, wealthy women have more than five times higher C-section use than poor women. In the United States, 32% of births were by C-section in 2015, an increase from 23% in 2000, as the data showed, and in the United Kingdom, 26.2% of births were by C-section in 2015, up from 19.7% in 2000. According to the World Health Organization report, the country with the lowest C-section rate, at 0.6% in 2010, was South Sudan and the country with the highest, at 58.1% in 2014, was the Dominican Republic. Whereas, some countries where more than half of births were by C-section were Brazil, at 55.5% in 2015; Egypt, at 55.5% in 2014; Turkey, at 53.1% in 2015; and Venezuela, at 52.4% in 2013 [41].

The overall prevalence of Caesarean section in Ethiopia was 29.55% (95% CI: 25.46–33.65). This report is higher than the study done in Saudi Arabia [42], Nigeria [43], Pakistan [44], India [5], Brazil [45] and low and middle-income countries analysis [46]. This discrepancy might be due to the age of the mother elapses the ideal birth time, significantly increasing, non-communicable disease, increasing electronic fetal monitoring availability and accessibility in referral and general hospitals. This study finding is lower than the study done in Nepal, North America and Western Europe, Latin America and the Caribbean [47]. This difference might be due to countries with a rich wealth index that may have the capacity to have modern operative obstetrics management as compared to low and middle countries. Hence, low and middle-income countries have resource limitation and c-section is resource-constrained, may have low comprehensive obstetric health care services.

Antepartum hemorrhage, non-reassuring fetal heart rate pattern, malpresentation, and malposition, failed induction, obstructed labor, multiple gestations, cephalopelvic disproportion were the most common indications of Caesarean section in Ethiopia. This study finding is supported by the study done in low and middle-income countries [46], Saudi Arabia [42], Ghana [6, 8], Jordan [4] and India [5].

Neonatal sepsis, stillbirth, prematurity, perinatal asphyxia, low Apgar score, and meconium aspiration syndrome were the most common neonatal complications following the Caesarean section in Ethiopia. This study finding is supported by the study done in India [5], Jordan [4], and Ghana [6].

Postpartum hemorrhage, surgical site infection, puerperal fever, anemia, and maternal mortality were the most common neonatal adverse outcome of Caesarean section in Ethiopia. The finding of this study is supported by the study done in India [5], Jordan [4], and African countries [48].

## Conclusion

In this study, the overall pooled prevalence of Caesarean section in Ethiopia was high. Non-reassuring fetal heart rate patterns, cephalopelvic disproportion, and obstructed labor were the most common indication of Caesarean section. Low Apgar score, perinatal asphyxia, and neonatal sepsis were the most common complication of neonates whereas postpartum hemorrhage and febrile morbidity were the common maternal complications following the Caesarean section in Ethiopia. Therefore, based on the study findings, the authors recommend a particular emphasis to follow the WHO recommendations and guidelines. Avoiding unjustified and unnecessary indications for Caesarean sections has a significantly higher impact to prevent poor maternal and fetal outcomes.

## Data Availability

All related data has been presented within the manuscript. The dataset supporting the conclusions of this article is available from the corresponding author on request.

## Abbreviations

CS: Caesarean section
PPH: Postpartum hemorrhage,
OL: Obstructed labor
CPD: Cephalopelvic disproportion
APH: Antepartum hemorrhage
SNNPR: South Nation Nationalities and Peoples region
CI: Confidence Interval
OR: Odd Ratio

## References

[1] Lyell DJ, Power M, Murtough K, Ness A, Anderson B, Erickson K, Schulkin J (2016). Surgical techniques at cesarean delivery: a US survey. Surg J.

[2] Strom S (2013). Rates, Trends, and Determinants of Cesarean Section Deliveries in El Salvador: 1998 to 2008 (doctoral dissertation).

[3] Rayburn WF, Strunk AL (2013). Profiles about practice settings of American College of Obstetricians and Gynecologists fellows. Obstet Gynecol.

[4] BĂƟĞŚĂ AM, Al-Daradkah SA, Khader YS, Basha A, Sabet F (2017). Cesarean ^ĞcƟŽn͗ incidence, causes, associated factors and outcomes: a NĂƟŽnĂů WrŽƐƉĞcƟǀĞ study from Jordan. Gynecol Obstet Case Rep.

[5] Desai G, Anand A, Modi D, Shah S, Shah K, Shah A (2017). Rates, indications, and outcomes of caesarean section deliveries: a comparison of tribal and non-tribal women in Gujarat, India. PLoS One.

[6] Gulati D, Hjelde GI. Indications for Cesarean Sections at Korle Bu Teaching Hospital. Ghana (Master's thesis); 2012.

[7] Betrán AP, Merialdi M, Lauer JA, Bing-Shun W, Thomas J, Van Look P, Wagner M (2007). Rates of caesarean section: analysis of global, regional and national estimates. Paediatr Perinat Epidemiol.

[8] Faith A (2019). Indication and predictors for caesarean sections in Ghana and the birth outcomes. Euro J Obstetrics Gynecology Reprod Biology.

[9] Central Statistical Agency (CSA) [Ethiopia] and ICF. Ethiopia demographic and health survey 2016: Addis Ababa, Ethiopia, and Rockville, Maryland, USA: CSA and ICF; 2016. available at: https://dhsprogram.com/pubs/pdf/FR328/FR328.pdf, accessed Mar 2020.

[10] Bailey P, Lobis S, Maine D, Fortney JA. Monitoring emergency obstetric care: a handbook: World Health Organization; 2009..

[11] Peters M, Godfrey C, McInerney P, Soares C, Hanan K, Parker D (2015). The Joanna Briggs institute Reviewers' manual 2015: methodology for JBI scoping reviews.

[12] Akki JS, Gemeda DH, Akessa GM (2015). A review of caesarean delivery in Southwest Ethiopia: incidence, indications, and outcomes. Afr J Midwifery Womens Health.

[13] Ayano B, Guto A (2018). Indications and outcomes of emergency caesarean section at St Paul's hospital medical college, Addis Ababa, Ethiopia 2017:(afoul month retrospective cohort study). Gynecol Reprod Health.

[14] Egger M, Smith GD, Schneider M, Minder C (1997). Bias in meta-analysis detected by a simple, graphical test. BMJ..

[15] Ioannidis J (2008). Interpretation of tests of heterogeneity and bias in metaanalysis. J Eval Clin Pract.

[16] Higgins J, Thompson SG (2002). Quantifying heterogeneity in a meta-analysis. Stat Med.

[17] Borenstein M, Hedges LV, Higgins JP, Rothstein HR. A basic introduction to fixed-effect and random-effects models for meta-analysis. Res Synth Methods. 2010;1(2):97–111. 10.1002/jrsm.12 26061376

[18] Tadesse H, Gessessew A, Medhanyie AA (2019). Trends and outcomes of cesarean delivery in Ayder comprehensive specialized hospital, Mekelle City, northern Ethiopia. East Afr J Health Sci.

[19] Tesfaye T, Hailu D, Mekonnen N, Tesfaye R (2017). Magnitude of maternal complication and associated factors among mothers undergone cesarean section at Yirgalem general hospital, SNNPR, Ethiopia. Risk.

[20] Tenaw Z, Kassa ZY, Kassahun G, Ayenew A (2019). Maternal Preference, Mode of Delivery and Associated Factors among Women Who Gave Birth at Public and Private Hospitals in Hawassa City, Southern Ethiopia. Ann Global Health.

[21] Solomon AA. Prevalence of Ceserean Section and Associated Factors in University of Gondar Comprehensive Referal Hospital, North West Ethiopia. BMC Res Notes Rev. 2019. 10.21203/rs.2.13345/v1.

[22] Hailu A (2016). Assessment of leading indication and outcome of cesarean delivery in Arba Minch general hospital- a Cross-Sectional Study (MSc dissertation, Arba Minch Universty).

[23] Olanipekun A (2017). Prevalence of caesarean section and the associated factors in private hospitals in Addis Ababa-a cross-sectional study (Doctoral dissertation, Addis Abeba Universty).

[24] Gutema H, Shimye A (2014). Cesarean section and associated factors at mizan aman general hospital, Southwest Ethiopia. J Gynecol Obstet.

[25] Yenit MK, Gezahegn T, Adefires M, Shiferaw AM. Cesarean section rate, maternal and fetal outcome of birth following cesarean section at Finoteselam hospital, Northwest Ethiopia: A Descriptive Retrospective Data. Glob J Med Res. 2016.

[26] Bago BJ (2018). Prevalence and its associated factors among women undergone operative delivery at Hawassa University comprehensive specialized hospital, southern Ethiopia, 2017. Gynecol Obstet.

[27] Abebe FE (2016). Factors leading to cesarean section delivery at Felegehiwot referral hospital, Northwest Ethiopia: a retrospective record review. Reprod Health.

[28] Tsegaye H (2017). Prevalence of caesarean section and associated factors in Addis Ababa hospitals, Addis Ababa, Ethiopia, 2017 (Doctoral dissertation, Addis Ababa University).

[29] Getahun A (2015). Outcome of cesarean section and the associated factors at jugel hospital, harari region, eastern Ethiopia. 2015(doctoral dissertation, Haramaya University).

[30] Bayou YT, Mashalla YJ, Thupayagale-Tshweneagae G (2016). Patterns of caesarean-section delivery in Addis Ababa, Ethiopia. Afr J Primary Health Care Fam Med.

[31] Wondie AG, Zeleke AA, Yenus H, Tessema GA (2019). Cesarean delivery among women who gave birth in Dessie town hospitals, Northeast Ethiopia. PLoS One.

[32] Tsega F, Mengistie B, Dessie Y, Mengesha M (2015). Prevalence of cesarean section in urban health facilities and associated factors in eastern Ethiopia: hospital based cross sectional study. J Preg Child Health.

[33] Geremew A (2015). Prevalence and Outcome of Caesarean Section in Attat Hospital, Gurage Zone, SNNPR, Ethiopia. Arch Med.

[34] Worku S (2016). Rate and associated factors of caesarean section at chirozonal hospital,west harergae, oromia regional state, eastern Ethiopia. (MSc dissertation, Haramaya University).

[35] Taye A, Yuya M (2015). One year retrospective analysis of prevalence of caesarean section in Jimma University specialized hospital, South Western Ethiopia. J Preg Child Health.

[36] Mengesha MB (2019). Maternal and fetal outcomes of cesarean delivery and factors associated with its unfavorable management outcomes; in Ayder Specialized Comprehensive Hospital, Mekelle, Tigray, Ethiopia. 2017 BMC Res Notes.

[37] Gebre S, Negasi A, Hailu A (2017). Criteria based clinical audit of cesarean section in a general Hospital in West Tigray, Ethiopia. J Women's Health Care.

[38] Melese A (2019). Magnitude of cesarean section delivery and its associated factors among mothers who gave birth at public hospitals in north wollo zone, northern Ethiopia. (Msc dissertation, Haramaya University).

[39] Kyu HH, Shannon HS, Georgiades K, Boyle MH (2013). Caesarean delivery and neonatal mortality rates in 46 low-and middle-income countries: a propensity-score matching and meta-analysis of demographic and health survey data. Int J Epidemiol.

[40] Souza JP, Gulmezoglu A, Lumbiganon P, Laopaiboon M, Carroli G, Fawole B (2010). Caesarean section without medical indications is associated with an increased risk of adverse short-term maternal outcomes: the 2004-2008 WHO global survey on maternal and perinatal health. BMC Med.

[41] Boerma T, Ronsmans C, Melesse DY, Barros AJ, Barros FC, Juan L, Moller AB, Say L, Hosseinpoor AR, Yi M, Neto DDLR (2018). Global epidemiology of use of and disparities in caesarean sections. Lancet.

[42] Zakai Ghadeer H, Alrowithi Abdullah S, Buhlaigah Afnan M, Alharbi Abdullah A, Hakami Abrar H, Alqahtani Hanoof A (2018). Prevalence of caesarean section and its indicating factors among pregnant women attending delivery at king Abdulaziz University hospital, Jeddah city during 2016. EC Gynaecol.

[43] Adewuyi EO, Auta A, Khanal V (2019). Cesarean delivery in Nigeria: prevalence and associated factors—a population-based crosssectional study. BMJ Open.

[44] Amjad A, Amjad U, Zakar R, Usman A, Zakar MZ, Fischer F (2018). Factors associated with caesarean deliveries among child-bearing women in Pakistan: secondary analysis of data from the demographic and health survey, 2012–13. BMC Pregnancy Childbirth.

[45] Almeida SD, Bettiol H, Barbieri MA, Silva AA, Ribeiro VS (2008). Significant differences in cesarean section rates between a private and a public hospital in Brazil. Cadernos de saude publica.

[46] Ojo VA, Okwerekwu FO (1988). A critical analysis of the rates and indications for caesarean section in a developing country. Asia-Oceania J Obstetrics Gynaecology.

[47] C-section deliveries nearly doubled worldwide since 2000, study finds. https://edition.cnn.com › c-section-rates-study-parenting-without-borders-intl.

[48] Esterhuizen TM (2019). Maternal and neonatal outcomes after caesarean delivery in the African surgical outcomes study: a 7-day prospective observational cohort study. Lancet Glob Health.

